# Elucidation of the roles of brown and brite fat genes: GPR120 is a modulator of brown adipose tissue function

**DOI:** 10.1113/EP087877

**Published:** 2020-03-29

**Authors:** Mark Christian

**Affiliations:** ^1^ School of Science and Technology Nottingham Trent University Clifton Nottingham NG11 8NS UK

## Abstract

**New Findings:**

- **What is the topic of this review?**
  Activation of brown adipose tissue with G protein‐coupled receptors as key druggable targets as a strategy to increase energy consumption and reduce fat mass.
- **What advances does it highlight?**
  GPR120 is a fatty acid receptor highly expressed in brown adipose tissue. Its activation by selective ligands increases brown adipose tissue activity. This is mediated by changes in mitochondrial dynamics resulting in increased O_2_ consumption leading to enhanced nutrient uptake and a reduction in fat mass.

**Abstract:**

The identification of druggable targets to stimulate brown adipose tissue (BAT) is a strategy to combat obesity due to this highly metabolically active tissue utilising thermogenesis to burn fat. Upon cold exposure BAT is activated by the sympathetic nervous system via β_3_‐adrenergic receptors. Determination of additional receptors expressed by brown, white and brite (brown‐in‐white) fat can lead to new pharmacological treatments to activate BAT. GPR120 is a G protein‐coupled fatty acid receptor that is highly expressed in BAT and further increases in response to cold. Activation of this receptor with the selective agonist TUG‐891 acutely increases fat oxidation and reduces fat mass in mice. The effects are coincident with increased BAT activity and enhanced nutrient uptake. TUG‐891 stimulation of brown adipocytes induces intracellular Ca^2+^ release which results in elevated O_2_ consumption as well as mitochondrial depolarisation and fission. Thus, activation of GPR120 in BAT with ligands such as TUG‐891 is a promising strategy to increase fat consumption.

## Graphical abstract

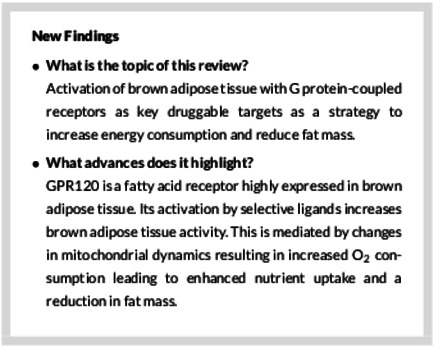

## 1 INTRODUCTION

Brown adipose tissue (BAT) possesses the unique ability to generate heat and dissipate energy through uncoupling protein 1 (UCP1)‐dependent thermogenesis. It is present and active in human adults and contributes to total energy expenditure (Cypess et al., 2009; van Marken Lichtenbelt et al., 2009; Virtanen et al., 2009). This contrasts with white adipose tissue (WAT), which is the main site of energy storage. The lipid‐storing cells in adipose tissue are the adipocytes, and there are a number of fundamental differences between brown and white adipocytes that determine their distinct functions. Brown adipocytes contain a large number of mitochondria and many small multilocular lipid droplets whereas white adipocytes have few mitochondria and store triacylglycerol (TG) in a single large unilocular lipid droplet (Cannon & Nedergaard, 2004; Cinti, 2012). There is a third type of adipocyte termed brite (brown‐in‐white) or beige which is found within WAT under conditions such as prolonged cold exposure (Wu et al., 2012). These cells are morphologically and functionally similar to classical brown adipocytes although they are derived from a different cellular lineage and have the ability to reversibly transition between white and brite‐type adipocytes (Rosenwald & Wolfrum, 2014).

The activation of brown and brite adipocytes initiates signal transduction pathways that result in the breakdown of TG to release fatty acids and glycerol. The fatty acids are oxidised in the mitochondria or allosterically activate UCP1, which is present on the inner mitochondrial membrane (Nicholls, 2017). UCP1 dissipates the proton gradient, resulting in the release of energy as heat rather than ATP production (Trayhurn, 2017). As activated BAT burns high amounts of fatty acids it is an important focus of researchers aiming to identify new strategies to combat obesity. The key signalling pathway for activation of BAT is through the β_3_‐adrenergic receptor (β_3_‐AR), which is simulated by noradrenaline released by the sympathetic nervous system in response to cold exposure (Argyropoulos & Harper, 2002). The strategies to find novel activators of BAT include screening of pharmacological compounds (Qiu et al., 2018), testing of individual candidate compounds or identification of the receptors expressed by brown/brite adipocytes.

### 1.1 GPR120

A receptor with a role in the control of brown adipocyte function is the G protein‐coupled receptor GPR120. It was identified as a potential target following analysis of the genes differentially expressed between brown and white adipose tissue alongside genes that are increased following cold exposure (Rosell et al., 2014). It is highly expressed in BAT, and following cold exposure it is increased in both the BAT and subcutaneous WAT of mice. These data indicate that this receptor could impact on the thermogenic capacity of BAT. GPR120/free fatty acid receptor (FFAR) 4 is important in the investigation of BAT function as it is a nutrient‐sensing receptor activated by fatty acids which are released by lipolysis. It is one of several GPCRs that are activated by fatty acids and that include GPR43/FFAR2, GPR41/FFAR3 and GPR42, which are activated by short‐chain fatty acids. GPR120/FFAR4 and GPR40/FFAR1 are activated by medium‐ and/or long‐chain fatty acids (Ichimura, Hasegawa, Kasubuchi, & Kimura, 2014; Puhl, Won, Lu, & Ikeda, 2015). GPR120 is of particular interest as it mediates anti‐inflammatory actions of ω‐3 polyunsaturated fatty acids and chronic inflammation is a key contributor to adipose tissue dysfunction (Oh et al., 2010).

Studies have revealed that mice deficient in GPR120 are more prone to developmental obesity and metabolic disorders including glucose intolerance and fatty liver (Ichimura et al., 2012). The insulin resistance in *Gpr120* knockout mice is associated with reduced insulin signalling and increased inflammation in adipose tissue. Furthermore, in the BAT of mice lacking *Gpr120*, UCP1 expression is reduced along with plasma fibroblast growth factor 21 (FGF21) levels, which contributes to defective thermogenesis (Quesada‐López et al., 2019). Investigations in humans have shown that WAT of obese individuals expresses higher levels of GPR120 compared to those of lean individuals (Ichimura et al., 2012). Furthermore, a non‐synonymous mutation in GPR120 (p.R270H) inhibits receptor signalling and is associated with increased risk of obesity in European populations.

### 1.2 GPR120 action in adipogenesis

The effects of GPR120 have primarily been studied in white adipocytes and some of these effects may be linked to a defect in the process of adipogenesis when its expression is ablated. SiRNA‐mediated knockdown of GPR120 expression in 3T3‐L1 cells inhibits lipid droplet accumulation and the expression of the marker of mature adipocytes fatty acid binding protein 4 (FABP4) (Gotoh et al., 2007; Liu, Wang, Meng, Kuang, & Liu, 2012). GPR120 expression increases during brown adipocyte differentiation (Schilperoort et al., 2018). This has similarly been reported in other adipocyte models including 3T3‐L1 cells, and furthermore, the GPR120 agonist TUG‐891 has been found to promote differentiation (Song et al., 2016). Brown adipogenesis due to GPR120 activation may also involve alteration in the expression of miRNAs. The GPR120‐activating ligand GW9580 was found to enhance miR‐30b in brown pre‐adipocytes (Quesada‐López et al., 2016). This miRNA targets RIP140, which is a key repressor of the brown adipose tissue programme (Kiskinis et al., 2014; Leonardsson et al., 2004). Thus, GPR120 activation could lead to reduced levels of RIP140 and consequently increased expression of BAT‐associated genes. Caution should be given to interpretation of the effects of GPR120 activators on differentiation due to the potential of the ligands used to activate pathways independent of GPR120 including peroxisome proliferator‐activated receptor γ (PPARγ) and prostaglandin receptors, which are key to the process of adipogenesis. Because of these considerations, it is important that strategies such as ablation of the receptor are employed to validate GPR120‐dependent effects.

### 1.3 Role of GPR120 in adipose tissue browning

The browning of WAT *in vivo* following TUG‐891 treatment was indicated in gonadal WAT by the increased UCP1 immunohistochemical staining (Schilperoort et al., 2018) coincident with a reduction in adipocyte size. It is notable that GPR120 expression is induced by acute β_3_‐AR activation of brown adipocytes *in vitro* (Schilperoort et al., 2018) indicating that it may be subject to similar regulatory mechanisms as UCP1 in addition to serving an important role when thermogenesis is activated. Furthermore, *in vitro* stimuli that promote the expression of the brown fat phenotype in white adipocytes increase the expression of GPR120. This response is observed with *in vitro* subcutaneous white adipocytes treated with the PPARγ agonist rosiglitazone (Schilperoort et al., 2018) or 3T3‐L1 adipocytes incubated with troglitazone (Gotoh et al., 2007). Furthermore, the browning of subcutaneous WAT in response to a cold exposure for 7 days is impaired in *Gpr120* knockout mice (Quesada‐López et al., 2016). Activation of GPR120 induces the release of FGF21, a thermogenesis‐promoting hormone, and a reduction in its release in the knockout mice is key to the lack of browning response. The increases in GPR120 expression during cold exposure correlates with brown status of adipocytes as well as the state of thermogenesis activation and highlight the importance of the receptor in control of brown and brite fat metabolism.

### 1.4 GPR120 increases fat oxidation in BAT

The therapeutic action of GPR120 in BAT has been investigated by treatment with selective agonists and results in reduced body weight (Azevedo et al., 2016; Schilperoort et al., 2018). The ligand TUG‐891 acutely increases fat oxidation and reduces body weight and fat mass in mice (Schilperoort et al., 2018). The involvement of BAT in these processes was confirmed by the enhanced uptake of fatty acids from lipoprotein‐like particles by BAT, indicating an increased lipid combustion in BAT resulting in a higher need to take up lipids from the circulation. Alongside these responses was decreased brown adipocyte lipid content. Acute treatment with TUG‐891 also led to an increase in glucose uptake by BAT. The effect of GPR120 activation of glucose uptake is supported by studies with ligand GW9508 that found it increased uptake in 3T3‐L1 adipocytes (Oh et al., 2010). Thus, GPR120 activation has an important role in metabolic substrate availability to cells within BAT.

### 1.5 Activated GPR120 affects mitochondrial function

The control of mitochondrial function is key to activation of browning and thermogenesis. The level of O_2_ consumption in brown adipocytes is increased following treatment with TUG‐891. Importantly, ligand treatment acutely induced O_2_ consumption, an effect that was through GPR120‐dependent and ‐independent mechanisms. Furthermore, investigations with mitochondrial dyes sensitive to membrane potential revealed that TUG‐891 promotes mitochondrial membrane depolarisation. This occurred alongside increased mitochondrial fragmentation. As GPR120 affects several aspects of mitochondrial activity, it is important to determine the different signalling pathways that mediate the changes in mitochondrial function.

### 1.6 Signal transduction pathways activated by GPR120

**FIGURE 1:**
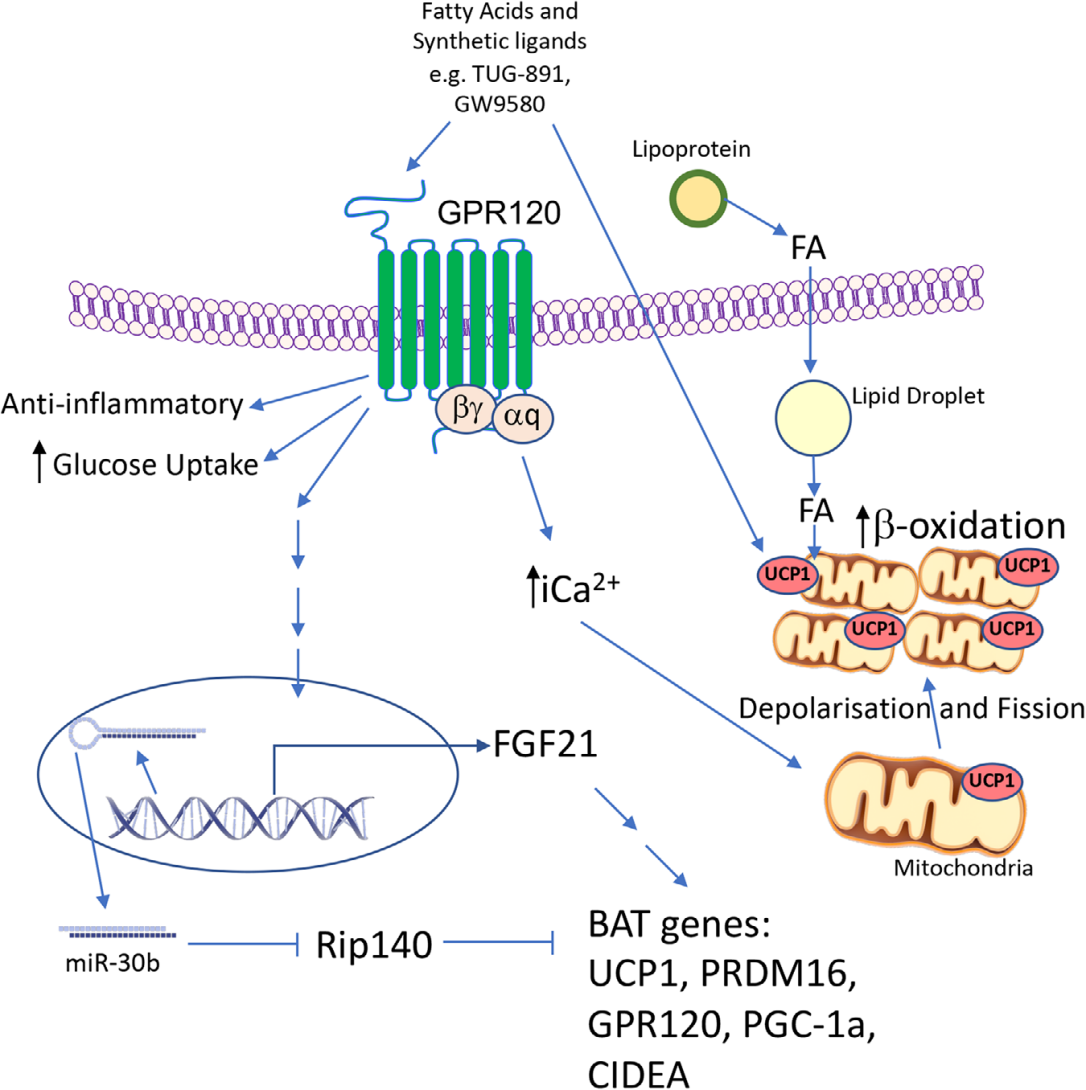
GPR120 actions in brown adipocytes. Fatty acids and synthetic ligands such as TUG‐891 activate the G_αq_‐coupled GPR120, which stimulates anti‐inflammatory pathways, glucose uptake and leads an increase in intracellular Ca^2+^ (iCa^2+^). Increased iCa^2+^ leads to depolarisation of the mitochondria, and mitochondrial fission, which increases respiration. Activated GPR120 results in increased expression of FGF21 and microRNAs including miR‐30b and results in the elevated expression of brown fat genes. In addition, mitochondrial UCP1 is directly activated by TUG‐891, further promoting uncoupled respiration and lipid combustion. This increases nutrient requirement and leads to uptake of fatty acids (FA) from circulating triglyceride (TG)‐rich lipoproteins. Together, these GPR120‐stimulated processes result in a reduction in fat mass

GPR120 activates a number of signal transduction pathways that have the capacity to modulate metabolic function in brown adipocytes (Figure 1). GPR120 is coupled to G_αq/11_, which stimulates intracellular Ca^2+^ release and phosphorylation events in the extracellular signal‐regulated kinases 1 and 2 (ERK1/2) cascade (Hudson et al., 2013). These pathways could affect the signalling events that control adipocyte differentiation. GPR120 also has been found to signal via AKT/nuclear factor‐κB in breast cancer cells to upregulate ABC transporters (Hudson et al., 2013). The contribution of these different pathways to mitochondrial activity in brown adipocytes was investigated using pathway‐selective inhibitors. Mechanistic *in vitro* investigations revealed that TUG‐891 treatment of brown adipocytes stimulates intracellular Ca^2+^ release and this was required for the increase in O_2_ consumption and was independent of the ERK and AKT pathways. The release of Ca^2+^ was dependent on G_αq_. The mechanism by which intracellular Ca^2+^ release promotes O_2_ consumption remains to be fully defined. There are a number of transporters that facilitate the entry of Ca^2+^ into the mitochondria. Increasing the Ca^2+^ levels in the mitochondria has the capacity to enhance the activity of mitochondrial dehydrogenases, enzymes essential for respiration. Transporters include the mitochondrial calcium uniporter and leucine zipper‐EF‐hand containing transmembrane protein 1 (LETM1), which interestingly was identified as cold‐induced in adipose tissues as well as being more highly expressed in BAT compared to WAT at thermoneutrality (Rosell et al., 2014). However, it is currently unclear whether LETM1 would increase or decrease the level of Ca^2+^ in brown adipocyte mitochondria. Furthermore, its activity may be subject to control by the proton gradient of the inner mitochondrial membrane (Shao et al., 2016).

### 1.7 GPR120 ligand activation of UCP1

GPR120 ligands can have off‐target effects and this raises the possibility of additional beneficial or detrimental actions. The *in vivo* actions of TUG‐891, although mostly through the receptor, have some effects in *Gpr120* knockout mice. Although the reduction of body weight due to TUG‐891 observed in wild type mice was not apparent in the knockout, there was an increase in the uptake of fatty acids from lipoprotein‐like particles in the receptor‐ablated mice. One of the GPR120‐independent effects found for TUG‐891 was a direct activation of UCP1. The action of the ligand was similar to that of oleate and other long‐chain fatty acids to relieve the natural GDP‐dependent inhibition of UCP1 (Fedorenko, Lishko, & Kirichok, 2012; Matthias et al., 2000; Shabalina, Jacobsson, Cannon, & Nedergaard, 2004) and thereby increase UCP1 activity and uncoupled mitochondrial respiration. Thus, TUG‐891 represents a GPR120 ligand with additional actions in mitochondria that are independent of the receptor.

## 2 CONCLUSION

Transcriptomic analysis to identify new receptors with the potential to activate brown adipocytes has been shown to be a valid approach. GPR120 activation enhances the metabolic activity of BAT and cultured brown adipocytes including nutrient uptake, O_2_ consumption and mitochondrial dynamics. The receptor is also expressed in white adipocytes and contributes to the conversion to brite adipocytes. As impaired signalling of GPR120 is associated with human obesity development, agonists for this receptor represent a promising strategy to reduce obesity by increasing lipid combustion. The potential of GPR120 ligands to have additional beneficial effects should also be considered as this receptor mediates anti‐inflammatory effects and the ligands could have off‐target effects such as direct activation of UCP1.

## COMPETING INTERESTS

The author declares no conflicts of interest.
